# Apigenin attenuates the atherosclerotic lesions through enhancing selective autophagy/lipophagy and promoting RCT process

**DOI:** 10.1080/13880209.2025.2509020

**Published:** 2025-05-23

**Authors:** Zixuan Hu, Yuting Li, Nan Yao, Haining Gan, Qiaohuang Zeng, Xuejun Huang, Dane Huang, Dake Cai, Yuxing Chen

**Affiliations:** a Guangdong Provincial Second Hospital of Traditional Chinese Medicine (Guangdong Provincial Engineering Technology Research Institute of Traditional Chinese Medicine), Guangzhou, China; b Guangdong Provincial Key Laboratory of Research and Development in Traditional Chinese Medicine, Guangzhou, China; c The Fifth Clinical College of Guangzhou, University of Chinese Medicine, Guangzhou, China; d School of Traditional Chinese Medicine and Health, Nanfang College · Guangzhou, Guangzhou, China

**Keywords:** Apigenin, dyslipidemia in atherosclerosis, selective autophagy/lipophagy, reverse cholesterol transport

## Abstract

**Context:**

Apigenin, a naturally flavonoid, is reported to have protective effects in chronic and metabolic diseases. But the therapeutic or ameliorative effects of apigenin on atherosclerosis are not known.

**Objective:**

Our study aimed to elucidate the underlying mechanism of apigenin on preventing atherosclerosis by enhancing selective autophagy/lipophagy and promoting RCT process.

**Materials and methods:**

ApoE^−/−^ mice fed with a high-fat diet (HFD) for 18 weeks were used to establish atherosclerosis model. Oil-Red-O staining of the plaques in the aorta and the heart was used to determine the severity of atherosclerosis. The autophagy flux was evaluated by western blot and reverse transcription quantitative PCR (RT-qPCR). Then triton WR-1339 (TWR) was injected into muscles of C57BL/6 mice, and the role of autophagy was assessed by autophagy inhibitor LY294002 intervention. The transmission electron microscopy (TEM) and immunofluorescence microscopy analysis (IFM) were used to elucidate the lipid-lowering mechanism of apigenin.

**Results:**

In HFD-induced mice, apigenin inhibited the dangerous progression of atherosclerosis through decreasing lipid deposition in plaques, lowering serum and liver lipid contents, activating autophagy and promoting reverse cholesterol transport (RCT). In TWR-induced mice, apigenin reduced the serum and liver lipid levels, enhanced the autophagy flux and increased RCT, but the above effects of apigenin were weakened by LY294002. The TEM and IFM images revealed that apigenin promoted the formation of autophagosomes and the co-localization between autophagy proteins with lipid protein.

**Discussion and conclusions:**

The lipid-lowering effects of apigenin were mediated through promoting RCT and enhancing selective lipophagy, meanwhile it provided a potential therapeutic option for atherosclerosis.

## Introduction

Atherosclerosis-related cardiovascular diseases are the leading causes of morbidity and mortality worldwide (Halasz et al. 2022). The accumulation and retention of lipid in the arterial intima leads to the functional impairment of vascular endothelial cells (Fan et al. 2021). Meanwhile, the lipid disorders and disturbance in coagulation aggravate the formation and instability of atherosclerotic plaques. The continuous progression of atherosclerosis leads to intra-plaque hemorrhagic regions, the plaque rupture, as well as the risk of thromboembolism and stroke (Li et al. 2023). Currently, despite some progress, questions regarding the pathogenesis of atherosclerosis remain and it is necessary to develop novel treatment strategies for atherosclerosis.

Recent studies have found that the pathological process of atherosclerosis is accompanied by the inhibition of autophagy in the early stage, and proper induction of autophagy is beneficial to reverse atherosclerosis progression (Zhang et al. 2021; Wang et al. 2022). Furthermore, Singh et al. have identified an autophagy-mediated lipolytic function that involves the lysosomal acid lipase A (LIPA) pathway, for which they have coined the term ‘lipophagy’ (Martinez-Lopez and Singh 2015). Lipophagy is a selective autophagy, which is the biological process that lipid droplets are sequestered by autophagosomes and eventually degraded by lysosomes (Laval and Ouimet 2023). Particularly intriguing is that researchers find the interaction between lipophagy and the turnover of lipid droplets (Liu et al. 2020). Therefore, promoting autophagy and regulating intracellular lipid droplets are potential strategies for preventing atherosclerosis.

Apigenin, a plant-derived flavonoid, is able to regulate lipid metabolism, inhibit cell inflammatory, and decrease platelet aggregation in chronic and metabolic diseases (Zhang et al. 2020; Jin et al. 2022). It has been shown that apigenin significantly reverses hepatic lipid accumulation and decreases body weight and plasma lipid levels in atherosclerotic mice (Lu et al. 2023). Although several studies have explored the effects of apigenin on autophagy mechanism, its effects on the promotion of lipid degradation and underlying mechanisms related to lipophagy have not been fully explored.

In this study, we aimed to explore the role and mechanisms of apigenin in autophagy and lipophagy, as well as its potential as a protective agent against atherosclerosis. Therefore, we fed a high-fat diet to apolipoprotein E knockout (ApoE^−/−^) mice for 18 weeks to establish atherosclerosis model and discovered that apigenin intervention significantly attenuated the atherosclerotic lesions. Furthermore, mechanistic studies were also performed to explore the effects of apigenin. We discovered that the protective effect of apigenin was associated with enhancing autophagy and lipophagy.

## Materials and methods

### Drugs

Apigenin was obtained from AmyJet Scientific Ltd (9B131955, pure ≥ 98%, Wuhan, China). Rosuvastatin was from Chengdu Herbpurify Ltd (Q-002-180131, Chengdu, China). Fenofibrate was bought from Abbott Laboratories Limited (27232, USA). Triton WR-1339 (TWR) was bought from Sigma-Aldrich Ltd (MKCC6730, Shanghai, China). LY294002 (2-Morpholino-8-phenylchromone, autophagy inhibitor) was from J&K Scientific Ltd (LT20Q192, pure ≥ 98%, Beijing, China).

### Animals

All animal experiments were reviewed and approved by the animal ethics committee of Guangdong Provincial Engineering Technology Research Institute of Traditional Chinese Medicine (No. 048610, 2018/03/29; No. 048603, 2018/03/15; No. 048592, 2018/01/23). Fifty ApoE^−/−^ mice (C57BL/6 background, 8 weeks old, male, 20 ∼ 25 g) and 10 C57BL/6 mice (8 weeks old, male, 20 ∼ 25 g) were purchased from Beijing Vital River Laboratory Animal Technology Co., Ltd. (Beijing, China) (No.11400700292598; No.11400700292599). A total of 120 C57BL/6 mice (8 weeks old, male, 20 ∼ 25 g) were provided by Guangdong Medical Laboratory Animal Center (No.44007200049468; No.44007200048236). All mice were housed in specific-pathogen-free laboratory at 22 ± 2 °C and 60% relative humidity. The experimental protocols involving mice were adhered to the ARRIVE guidelines.

### High-fat diet-induced animal model

ApoE is the major ligand for lipoprotein receptors whose function is to remove chylomicrons and lipoproteins (Figueiredo et al. 2022). The pathological characteristics of the ApoE-knockout mice with atherosclerosis are very similar to those of humans with atherosclerosis (Wang et al. 2021). Therefore, ApoE^−/−^ mice were used to establish atherosclerosis model.

After being habituated the environment for one week, 10 C57BL/6 mice were used as a normal control group (NC) and fed with a normal diet for 18 weeks. ApoE^−/−^ mice were divided into five groups (*n* = 10): high-fat diet group (HFD), 1.3 mg/kg/d rosuvastatin group (RST), 25 mg/kg/d apigenin group (APG-H), 12.5 mg/kg/d apigenin group (APG-M), 6.25 mg/kg/d apigenin group (APG-L). The dosage of apigenin used in this study was based on *Research Methods in Pharmacology of Chinese Material Medica* and pre-experiments results. ApoE^−/−^ mice were treated with high-fat diet (20% sucrose, 15% lard, 10% casein, 1.2% cholesterol, 52.2% basic feed) for 18 weeks. In the ninth week, the drugs were orally administrated to mice for nine weeks, respectively. At the end of the experiment, the mice were anesthetized with 0.41 mL/min isoflurane, blood was obtained by eyeball extirpation and care was taken to reduce animal suffering; then, the mice were euthanized under deep anesthesia (isoflurane) *via* cervical dislocation.

### Triton WR-1339-induced animal model

Hyperlipidemia is the material basis for the formation of atherosclerotic lesions. TWR-induced hyperlipidemia mice possess multiple pathological characteristics including over production of endogenous lipid, dysfunction of reverse cholesterol transport and hepatic acute injury (Li et al. 2020). Therefore, TWR-induced hyperlipidemia model by C57BL/6 mice was employed to validate the effect of apigenin activating autophagy toward disorder of endogenous lipid metabolism.

The C57BL/6 mice were fed normal diet and divided into six groups randomly (*n* = 10): normal control group (NC), triton WR-1339-treated group (TWR), TWR＋26 mg/kg/d fenofibrate group (FG), TWR＋25 mg/kg/d apigenin group (APG-H), TWR＋12.5 mg/kg/d apigenin group (APG-M), TWR＋6.25 mg/kg/d apigenin group (APG-L). Another C57BL/6 mice were fed normal diet and divided into six groups (*n* = 10): normal control group (NC), TWR model group (TWR), TWR＋26 mg/kg/d fenofibrate group (FG), TWR＋25 mg/kg/d apigenin group (APG), TWR＋1.5 mg/kg/d LY294002 group (LY), TWR＋1.5 mg/kg/d LY294002 + 25 mg/kg/d apigenin (APG+LY). The drugs were orally administered to mice for 5 days, respectively. At 9 pm on the third day, triton WR-1339 was injected at 0.12 g/mL to the mice. At 9 am on the fifth day, 1h following gavage, the mice were anesthetized with 0.41 mL/min isoflurane, blood was obtained by eyeball extirpation and care was taken to reduce animal suffering, then the mice were euthanized under deep anesthesia (isoflurane) *via* cervical dislocation.

### Histopathological analysis

The aortas and heart tissues were stained with Oil-Red-O Kit (20180413, NJJC Bio, Nanjing, China). The whole aortas were fixed with 4% paraformaldehyde for 24 h, then stained with Oil-Red-O liquid for 6 min and washed twice with 60% isopropanol (C16700105, MACKLIN, Heze, China). The pictures were taken with camera, and Image Pro Plus software was used to calculate plaque area/total vessel area. The heart tissues were fixed in OCT (Tissue-Tek), frozen in liquid nitrogen and sectioned into about 8 μm of sections on Leica frozen section machine. Then the sections were immobilized in 4% paraformaldehyde and washed with 60% isopropanol. Sections from the heart were soaked with Oil-Red-O solution and counterstained with hematoxylin at room temperature. The pictures were taken with microscope (Olympus DP72), and the area of oil droplets was analyzed semiquantitatively with Image Pro Plus software.

The liver tissues fixed with 10% paraformaldehyde (BIBM00105, Dingguo Changsheng Bio, Beijing, China) were made into paraffin sections with 4 ∼ 6 μm thickness. The sections were stained with hematoxylin and eosin (H&E) solution (BP-DL017, SenBeiJia Bio, Nanjing, China) for evaluating the hepatic histopathological changes. Finally, the slides were observed under the light microscope (Olympus, Japan). The semi-quantitative score system was used to score the liver tissues for four slices per group, hepatocellular fatty degeneration (0–3), lipid droplet (0–3), hepatocellular ballooning degeneration (0–3). Score was accorded to the grade of lesion, normal (0), slight (0.5), mild (1), moderate (2) and severe (3) (Feng et al. 2017).

### Enzyme-linked immunosorbent assay (ELISA)

Liver samples were cut into small pieces and weighed. Then, the tissues were homogenized in Trizol reagent (B0BL00110, Dingguo Changsheng, Beijing, China) and centrifuged at 2500 pm for 10 min at 4 ◦C. The supernatants obtained were transferred to a clean tube. Total cholesterol (TC, 20180722), triglyceride (TG, 20180822) and low-density cholesterol (LDL-C, 20180322) levels of liver were determined by using commercially available ELISA kits from Shanghai Kexin Biotechnology Research Institute (Shanghai, China) according to the manufacturer’s protocol.

### Biochemical analyses

After collecting blood from the mice, the blood was centrifuged at 3,000 rpm for 10 min to separate the serum. TC (20180122), TG (20180112) and LDL-C (20180911) levels were measured with a fully automated biochemical analyzer (Hitachi, Japan).

### Transmission electron microscopy (TEM)

Transmission electron microscopy was used to evaluate autophagosomes and lipid droplets in liver tissues. The liver tissues were fixed with 4% paraformaldehyde buffer to preserve the structure of cells and washed three times with PBS. Then the samples were postfixed in 1% osmium tetroxide for 2 h and dehydrated in a graded ethanol series. Subsequently, they were infiltrated an epoxy embedding medium at 37 °C for overnight, polymerized at 60 °C for 48 h, and sectioned. After stained with uranyl acetate and lead citrate, the images were obtained with TEM to observe the formation of autophagosomes. The instruments and materials used in the transmission electron microscope were from Wuhan Google Biotechnology Ltd.

### Immunofluorescence microscopy analysis (IFM)

To observe the co-localization of autophagy protein and lipid protein, the paraffin sections of liver tissues were made according to the conventional method. Then, the sections were infiltrated with 0.1% Triton-X-100 at room temperature for 15 min and blocked with 5% BSA for 1h. Subsequently, the sections were incubated with primary antibody overnight in 4 °C and fluorescent secondary antibody for 2h in room temperature. Finally, the sections were counterstained with DAPI for 15 min in the dark and observed under a laser scanning confocal microscope (Wuhan Google Biotechnology, Wuhan, China).

### Reverse transcription quantitative PCR (RT-qPCR)

Total RNA from cultured tissues was extracted by Trizol reagent and reverse transcribed to cDNA with the reverse transcript Kit (AK92068A, TaKaRa Bio, Kusatsu, Japan). Quantitative amplification was performed on iQ5 optical system (BIO-RAD, Hercules, USA) with One Step PrimeScript PCR Kit (AK51812A, TaKaRa Bio, Kusatsu, Japan). All samples were assayed in triplicate. The target genes were analyzed using the 2^-ΔΔCt^ method, with 18s as internal control in the data analysis. The primers used were listed in Table 1.

**Table 1.** The primer sequences for mRNA in RT-qPCR.

Primer name	Primer sequences
mBeclin-1	*Sense:* GGGTCTAAGGCGTC *Anti-sense:* CTGGGCTGTGGTAA
mAtg14	*Sense:* GCTCACCTCCATCATATTCCC *Anti-sense:* CATCACAGACCCATCTTCCAG
mAtg5	*Sense:* AGTCTGTCCTTCCGCA *Anti-sense:* GTCACGCCTCGTTGTC
mUvrag	*Sense:* TGGAAAGTCTACCTGGATGGG *Anti-sense:* TTCTGTGCGTTTGGATGACC
mLc3b	*Sense:* GCTAACCAAGCCTTCTTCCTCC *Anti-sense:* TTGCTGTCCCGAATGTCTCCTG
mCd36	*Sense:* CCTTACACATACAGAGTTC *Anti-sense:* CTACAGCCAGATTCAGA
mAbcg1	*Sense:* CCTGGGGATTGGGAACGAAG *Anti-sense:* AAGGTCAGAACGGTGGGCAT
mSr-bI	*Sense:* ACAGAGCGGAGCAATGGGT *Anti-sense:* AAGAAGCGGGGTGTAGGGA
m18s	*Sense:* ACGGCTACCACATCC *Anti-sense:* CAGACTTGCCCTCCA

### Western blot analysis

The liver tissues were homogenized in RIPA lysis buffer (BB50323, BestBio, Shanghai, China) at 4 °C and the supernatant was centrifuged. The bicinchoninic acid (BCA) assay (WH328951, Thermo Fisher Scientific, MA, USA) method was used to quantify proteins. Protein samples were separated by electrophoresis and transferred onto polyvinylidene fluoride (PVDF) membrane (0.45 μm; 0000179258, EMD Millipore, Billerica, MA, USA). The membrane was blocked with 5% skim milk (H805BA0006, Sangon Bio, Shanghai, China) and washed with TBST (T1082, Solarbio Bio, Beijing, China), and incubated overnight with primary antibodies at 4 °C. The following primary antibodies were used: β-actin (ab8226, 1:2000, Abcam, RRID: AB_3696461, UniProt ID: P60709), α-tubulin (ab7291, 1:5000, Abcam, RRID: AB_3696470, UniProt ID: P68366), ATP-binding cassette transporter 5 (ABCG5) (27722-1-AP, 1:1000, Proteintech, RRID: AB_2880952, UniProt ID: Q9H222), Beclin-1 (66665-1-Ig, 1:2000, Proteintech, RRID: AB_2882020, UniProt ID: Q14457), unc-51-like autophagy-activating kinase 1 (ULK1) (20986-1-AP, 1:1000, Proteintech, RRID: AB_2878783, UniProt ID: O75385), UVRAG (19571-1-AP, 1:1000, Proteintech, RRID: AB_10640523, UniProt ID: Q9P2Y5), LC3 (18725-1-AP, 1:2000, Proteintech, RRID: AB_2137745, UniProt ID: Q9GZQ8), autophagy related protein 3 (ATG3) (11262-2-AP, 1:1000, Proteintech, RRID: AB_2059234, UniProt ID: Q9NT62), autophagy related protein 5 (ATG5) (10181-2-AP, 1:2000, Proteintech, RRID: AB_2062045, UniProt ID: Q9H1Y0), scavenger receptor class B type I (SR-BI) (ab217318, 1:2000, Abcam, RRID: AB_3696478, UniProt ID: Q8WTV0). Next, blots were washed thrice and probed using Goat anti-Rabbit IgG secondary antibodies (ab97051, 1:10000, Abcam, RRID: AB_10679369) or Mouse (ab97023, 1:10000, Abcam, RRID: AB_10679675) for 2 h. Finally, chemiluminescence was used to visualize protein bands (5220 Multi, Tanon; Shanghai, China).

### Statistical analysis

All the grouped data were statistically evaluated with the SPSS 22.0 software. The difference among several means was analyzed by using one-way ANOVA (LSD or Dannet’s T3). And *p* < 0.05 was considered to be statistically significant. Data were expressed as mean ± SD in excel software.

## Results

### Apigenin alleviated atherosclerosis in ApoE^−/−^ mice

**Figure 1.**
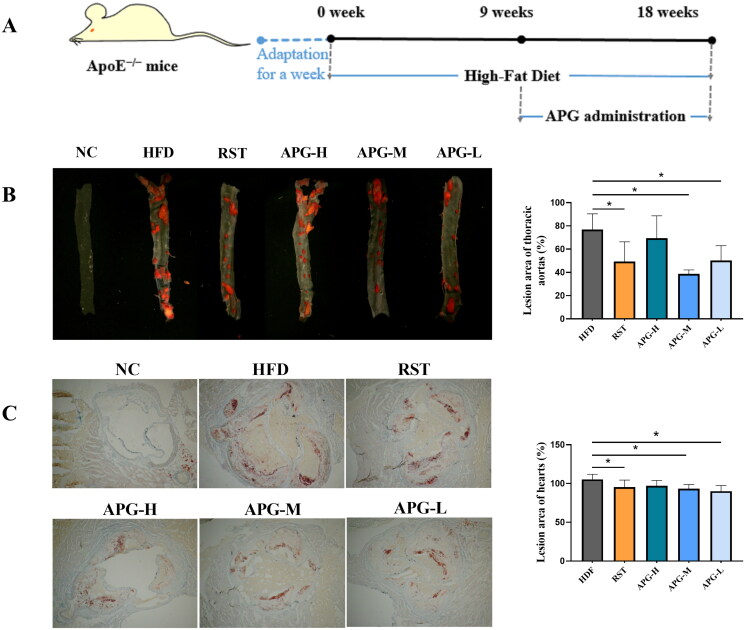
Apigenin alleviated the atherosclerotic lesions in ApoE^−/−^ mice. (A) Flowchart of HFD induced animal model. (B) Histology of oil Red O staining of thoracic aorta lesion (left). Atherosclerotic plaques were visualized by red color. Quantification analyses of atherosclerotic plaque of thoracic aorta lesion was shown in the right (*n* = 6). (C) Histology of oil Red O-stained coronal sections of heart (left). Quantification analyses of coronal sections in heart (*n* = 6). *p < 0.05 and **p < 0.01.

We evaluated the inhibitory effect of apigenin on the progression of atherosclerosis in ApoE^−/−^ mice by Oil-Red-O staining. Figure 1(A) presents a flowchart of HFD-induced animal model. Compared to those in the C57BL/6 mice, the aorta lesion and lipid accumulation were shown robust increases in HFD induced mice. The total area (%) of atherosclerotic plaques in the aorta revealed that RST, APG-M and APG-L groups all significantly reduced plaque areas the in HFD-fed mice (Figure 1(B)). Moreover, we also evaluated the therapeutic effect of apigenin against lipid deposition in the heart arteries. This suggested that apigenin, as well as rosuvastatin, could effectively reverse the increase of HFD-induced lipid content (Figure 1(C)). Altogether, apigenin could decrease lipid deposition in plaques and inhibit the dangerous progression of atherosclerosis.

### Apigenin effectively reduced lipid levels in ApoE^−/−^ mice

**Figure 2.**
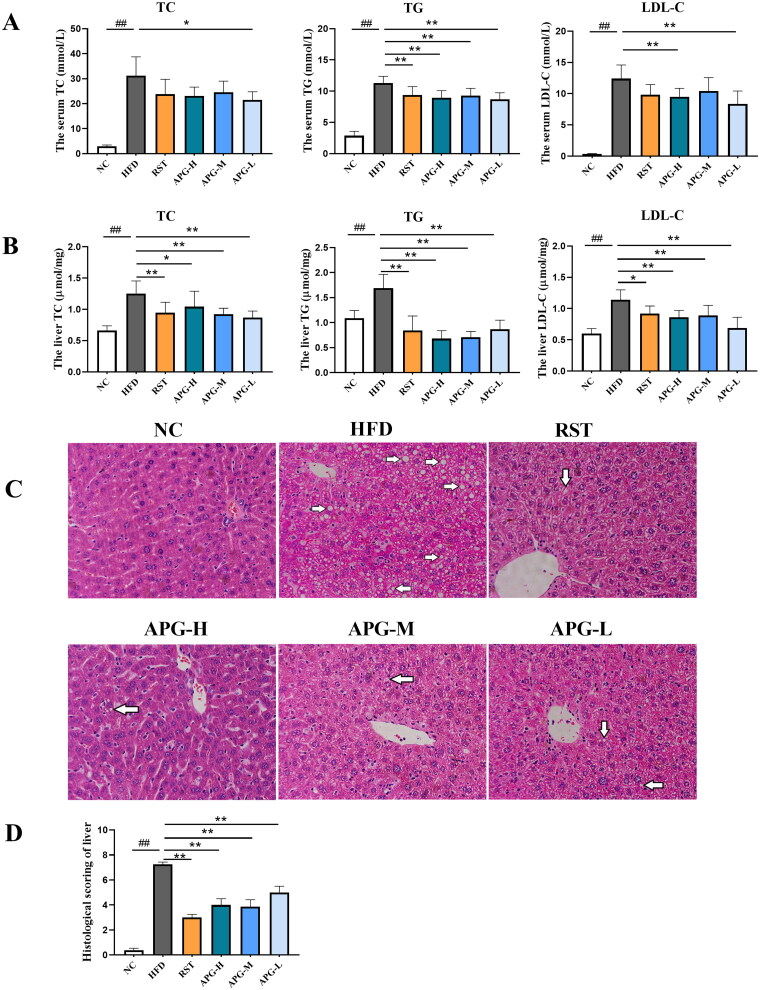
Apigenin reduced the lipid levels in HFD-induced mice. (A) Serum TC, TG and LDL-C concentrations (*n* = 10). (B) Liver TC, TG and LDL-C concentrations (*n* = 10). (C) The liver histopathology was analyzed by H&E staining (200× magnification). Morphological investigation includes observation of lipid droplet accumulation and hepatocellular ballooning degeneration (indicated by white arrow). (D) Histological scoring of liver (*n* = 4). #*p* < 0.05, ##*p* < 0.01 vs NC group; *p < 0.05 and **p < 0.01.

In order to investigate the biochemical basis that led to the pathological results, we collected samples from the ApoE^−/−^ mice to determine the serum and liver TC, TG and LDL-C levels. As shown in Figure 2(A), relative to HFD group, the levels of serum TG, TC and LDL-C were remarkably decreased by apigenin without dose-dependent effect. Moreover, the levels of liver TC, TG and LDL-C were significantly decreased in administration groups (Figure 2(B)). The date showed that apigenin could effectively reduce the serum and liver lipid levels in ApoE^−/−^ mice with a high-fat diet. We further analyzed pathological lesions in the liver tissue sections of ApoE^−/−^ mice and evaluated the severity of atherosclerosis based on H&E staining of the paraffin-embedded sections. As shown in Figure 2(C), the mice in NC group shown a regular hepatic structure and no steatosis. HFD group was observed the disorganization of hepatic structure, more hepatocytes appeared cytoplasmic vacuolation and large lipid droplet accumulation. By comparison, the accumulation of hepatocellular ballooning degeneration and lipid droplets were markedly decreased in apigenin and rosuvastatin-treated ApoE^−/−^ mice (Figure 2(D)). These data underlined the capacity of apigenin to alleviate dyslipidemia and reverse atherosclerosis *in vivo*.

### Apigenin activated and promoted autophagy in ApoE^−/−^ mice

**Figure 3.**
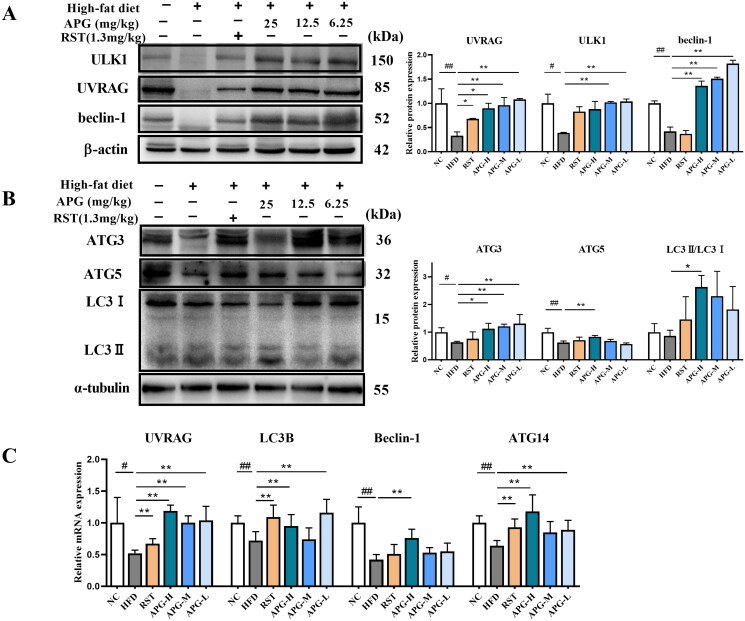
Apigenin activated and promoted autophagy in ApoE^−/−^ mice. (A) Western blot analysis of ULK1, UVRAG and beclin-1 were shown in left, and graph on the right represented the quantification analysis of autophagy proteins normalized by β-actin (*n* = 3). (B) Western blot analysis of ATG3, ATG5 and LC3 were shown in left, and graph on the right represented the quantification analysis of autophagy proteins normalized by α-tubulin (*n* = 3). (C) The mRNA expression levels of UVRAG, LC3B, Beclin-1 and ATG14 were examined by RT-qPCR (*n* = 10). #*p* < 0.05, ##*p* < 0.01 vs NC group; *p < 0.05 and **p < 0.01.

Based on the significance of autophagy to regulate lipid storage and metabolism (Yu et al. 2022), we speculated the anti-atherosclerosis property of apigenin was related to autophagy. The expression of autophagy-related proteins was examined by western blot. As shown in Figure 3(A), the protein expression of UVRAG, ULK1 and beclin-1 were markedly lower after HFD treatment alone as compared with NC group, subsequently the changes were rescued by apigenin administration the changes were. UVRAG was an important regulator of autophagy by interacting with Beclin1/PI3KC3 and ATGs, leading to activation of autophagy (Song et al. 2020). Apigenin also significantly increased the protein expression of ATG5 and ATG3 as compared with HFD group (Figure 3(B)). In our study, APG-H group increased the conversion of LC3-I to LC3-II as compared with HFD group in ApoE^−/−^ mice (Figure 3(B)), suggesting that apigenin promoted the completion of the autophagic flux. In addition, RT-qPCR was performed for determining the expression levels of autophagy-related mRNA. And apigenin remarkably increased the mRNA expression of UVRAG, LC3B, beclin-1 and ATG14 as compared with HFD group (Figure 3(C)). The results shown that apigenin might participate in the lipid metabolism progression through activating and promoting autophagy in ApoE^−/−^ mice.

### Apigenin facilitated RCT process in ApoE^−/−^ mice

**Figure 4.**
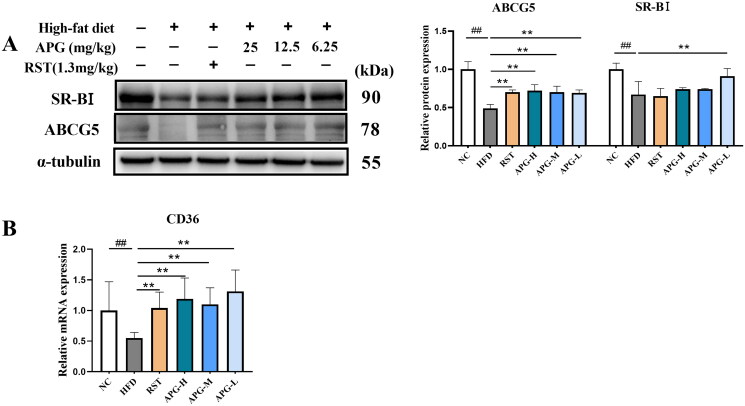
Apigenin facilitated RCT process in ApoE^−/−^ mice. (A) Western blot analysis of ABCG5 and SR-BI in ApoE^−/−^ mice were shown in left, and graph on the right represented the quantification analysis of the proteins normalized by α-tubulin (*n* = 3). (B) The mRNA expression level of CD36 was examined by RT-qPCR (*n* = 10). #*p* < 0.05, ##*p* < 0.01 vs NC group; *p < 0.05 and **p < 0.01.

The reverse cholesterol transport (RCT) is an important role in the development of atherosclerosis, by which the cholesterol moves out of foam cells in atherosclerotic plaques, enters the circulation, delivers to the liver, and is excreted in the feces (Ouimet et al. 2019). Herein, we investigated the effects of apigenin on CD36, ABCG5 and SR-BI expression in ApoE^−/−^ mice. As shown in Figure 4(A), apigenin remarkably increased the protein expression of ABCG5 and SR-BI as compared with HFD group. Moreover, the mRNA expression of CD36 was inhibited in ApoE^−/−^ mice treated with HFD alone, while it was notably increased in RST, APG-H, APG-M and APG-L groups (Figure 4(B)). In summary, apigenin contributed positively to RCT pathway in hepatic cells and increased the activities of cholesterol efflux.

### Apigenin effectively alleviated dyslipidemia in TWR-induced mice

**Figure 5.**
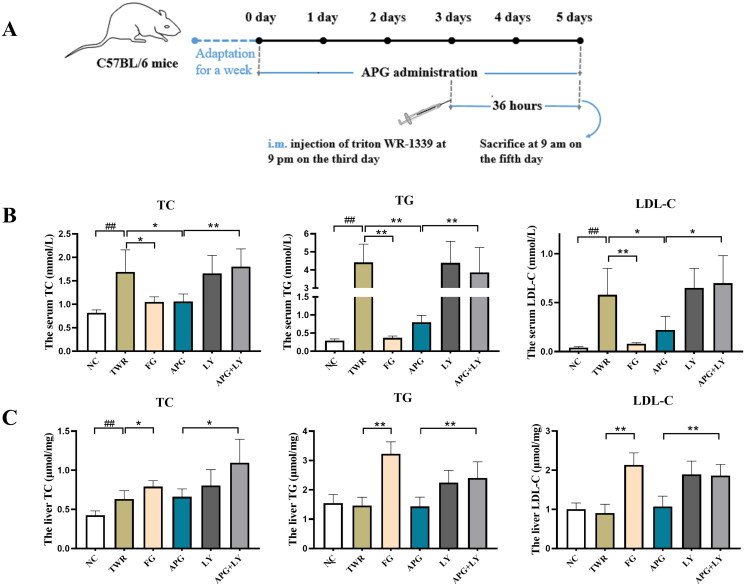
Apigenin alleviated lipid accumulation in TWR-induced mice. (A) Flowchart of TWR-induced animal model. (B) Serum TC, TG and LDL-C concentrations (*n* = 10). (C) Liver TC, TG and LDL-C concentrations (*n* = 10). #*p* < 0.05, ##*p* < 0.01 vs NC group; *p < 0.05 and **p < 0.01.

**Figure 6.**
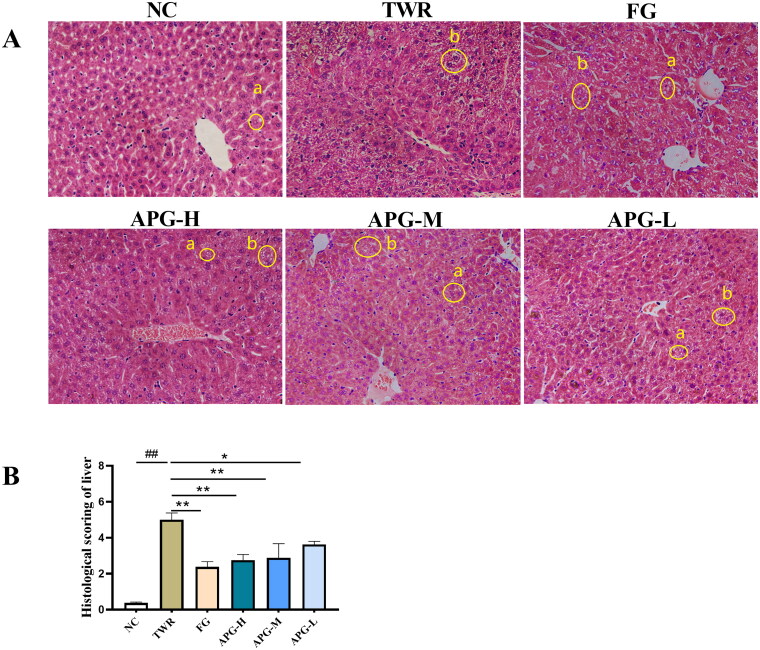
Apigenin alleviated lipid accumulation in TWR-induced mice. (A) The liver histopathology was analyzed by H&E staining in C57BL/6 mice (200× magnification). Morphological investigation includes observation of lipid droplet accumulation (a) and hepatocellular ballooning degeneration (b). (B) Histological scoring of liver (*n* = 4). #*p* < 0.05, ##*p* < 0.01 vs NC group; *p < 0.05 and **p < 0.01.

TWR is a nonionic detergent bind triglyceride-rich lipoproteins (Zhou et al. 2022), and it could disrupt lipid metabolism in mice by inhibiting lipoprotein lipase activity and stimulating the activity of 3-hydroxy-3-methyl glutaryl coenzyme A reductase (HMGCR) in liver (Bouhlali et al. 2020; Guo et al. 2021). To further investigate the hypolipidemic efficacy of apigenin on the molecular mechanisms, TWR was injected into the muscles of mice to establish acute hyperlipidemia model (Figure 5(A)). As shown in Figure 5(B), an obviously increase of serum TC, TG and LDL-C was observed in TWR group. In contrast, apigenin remarkably decreased the level of serum TC, TG and LDL. While the lipid-lowering effect of apigenin was reversed after the C57BL/6 mice were treated with the autophagy inhibitor LY294002, suggesting that autophagy participated in the regulation of lipid metabolism by apigenin. We further analyzed the liver lipid levels in C57BL/6 mice. As shown in Figure 5(C), liver TC was significantly increased in TWR group, while TG and LDL-C levels did not show significant difference as compared with NC group. It has reported that this increase in hepatic TG is not observed when assessed over longer time intervals after TWR injection appears to be due to the decrease in the hepatic TG production rate (Millar et al. 2005). In contrast, liver TC, TG and LDL-C levels were abnormally increased by fenofibrate administration. Meanwhile, H&E staining showed that TWR-induced hepatocellular ballooning degeneration and irregular central vein congestion, while they were markedly alleviated by the treatment of apigenin (Figure 6). These results demonstrated that apigenin effectively alleviated dyslipidemia in TWR-induced mice, which might be realized through autophagy activity.

### Apigenin promoted the formation of autophagosomes

**Figure 7.**
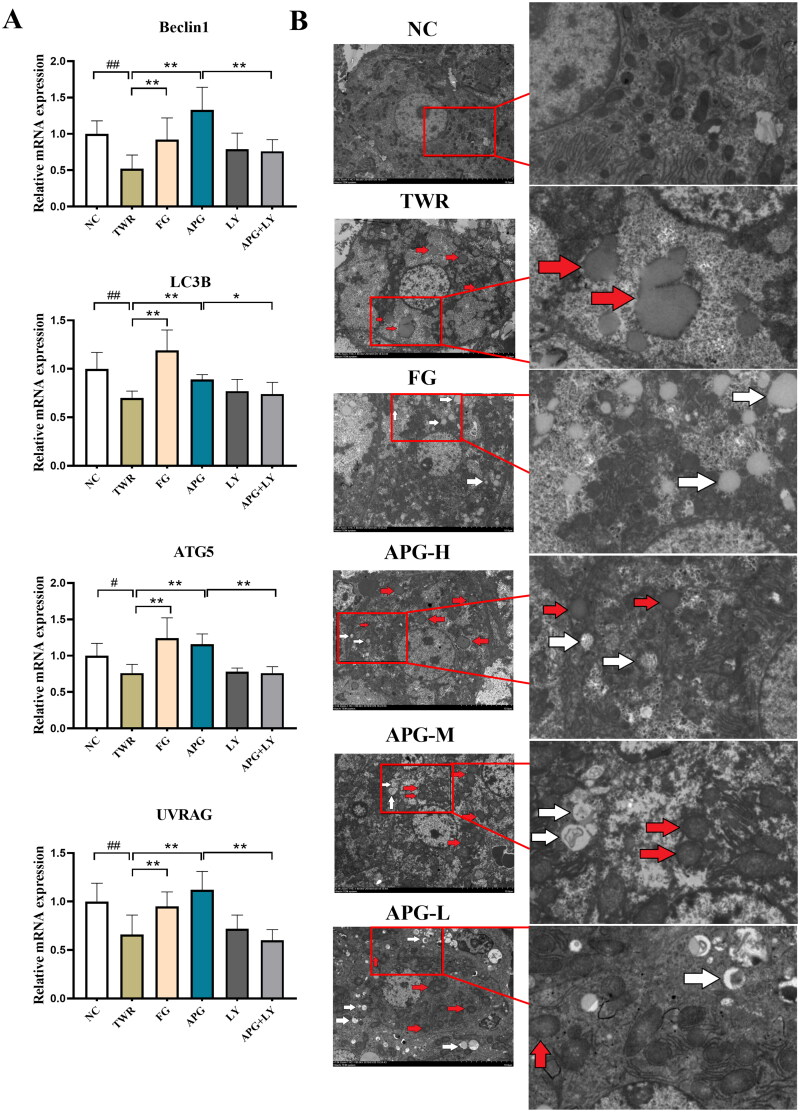
Apigenin promoted the formation of autophagosomes in TWR-induced mice. (A) The mRNA expression levels of UVRAG, LC3B, Beclin-1 and ATG5 were examined by RT-qPCR. (B) Transmission electron microscope images of hepatocyte in C57BL/6 mice (10 μm for the left images, 2 μm for the right images). Enlarged inserts at the right side of each image show lipid drop or damage organelle (red arrow), autophagosome (white arrow). #*p* < 0.05, ##*p* < 0.01vs NC group; *p < 0.05 and **p < 0.01.

Subsequently, to explore the impact of apigenin on hepatocyte autophagy, four autophagy-related genes were measured using PCR method. Relative to TWR groups, apigenin increased the expression of ULK1, UVRAG, ATG5 and LC3B, suggesting that apigenin activated autophagy (Figure 7(A)). And we performed PI3K inhibitor LY294002 to study whether activated autophagy could be reversed. Relative to APG group, LY294002 suppressed the mRNA expression of ULK1, UVRAG, ATG5 and LC3B and impeded apigenin-induced autophagy flux (Figure 7(A)). Next, we utilized TEM to assess the ultrastructural changes in hepatocytes. The TEM scanning revealed that TWR induced more lipid droplets and damaged organelles in comparison with NC group. However, apigenin treatment markedly increased autophagosome accumulation and decreased damaged organelles (Figure 7(B)). These results indicated that apigenin could enhanced the autophagy flux and promote the formation of autophagosomes.

### Lipophagy was involved in the inhibition of lipid accumulation by apigenin

**Figure 8.**
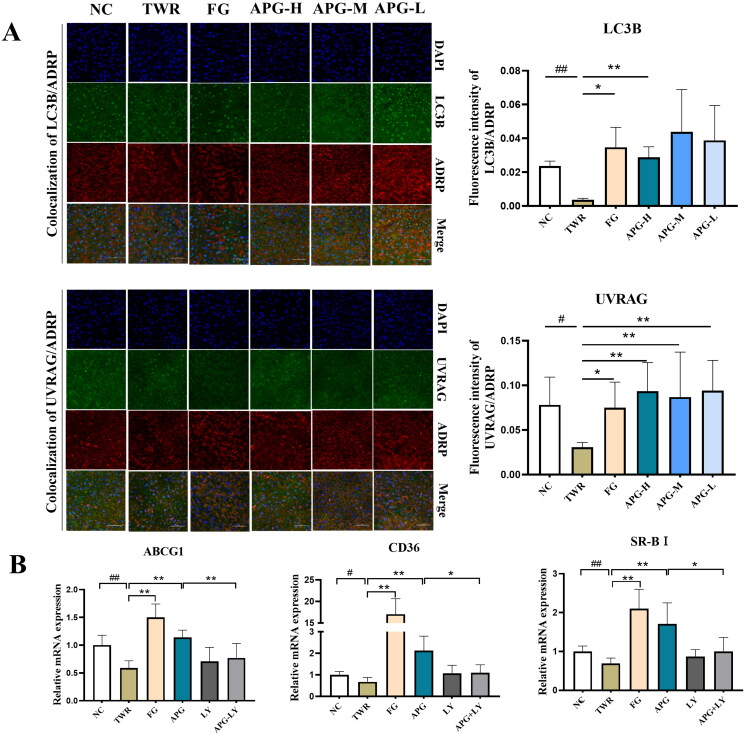
Apigenin promoted RCT process and inducted the co-localization of UVRAG/LC3B and ADRP. (A) The co-localization of LC3B/ADRP and UVRAG/ADRP under a confocal microscope (left). Quantification analyses of the co-localization of autophagy protein and lipid protein were shown in the right. (B) The expressions of ABCG1, CD36 and SR-BI were examined by RT-qPCR (*n* = 10). #*p* < 0.05, ##*p* < 0.01 vs NC group; *p < 0.05 and **p < 0.01.

Cholesterol efflux is the initial step of RCT, and numerous studies have shown that the regulation of autophagy and the process of cholesterol efflux is often correlated (Wang et al. 2019; Guo et al. 2022). Then we assessed whether autophagy was involved in apigenin-induced cholesterol efflux. Compared with TWR group, the expressions of cholesterol transport genes CD36, ABCG1 and SR-BI were significantly up-regulated by apigenin. While the autophagy inhibitor LY294002 suppressed these changes, and impeded apigenin-induced cholesterol efflux (Figure 8(B)). Lipophagy, as a selective autophagy, had been reported to specifically target and clear excess lipid in the liver (Minami et al. 2023). We observed the co-localization of autophagy related proteins (UVRAG, LC3B) and adipocyte differentiation-related protein (ADRP) in hepatocytes by scanning confocal microscopy (Figure 8(A)). Quantitative analyses demonstrated that the expression of UVRAG and LC3B (green fluorescence) in the administration groups had an upward tendency, while it had no statistics difference (*p* > 0.05) as compared with TWR group. Interestingly, autophagy proteins co-localized with the lipid marker ADRP had a significant increase in orange fluorescent dots present in the cytoplasm, indicating that apigenin inducted selective autophagy/lipophagy by regulating the cellular localization (Figure 8(A)). Collectively, apigenin enhanced cholesterol efflux and reduced lipid accumulation by activating UVRAG/LC3B-dependent selective lipophagy.

## Discussion

In this study, atherosclerosis model and hyperlipidemia model were used to explore unknown mechanism of apigenin regulating lipid metabolism in regard with pharmacodynamics and pathological diversity. It was shown that apigenin inhibited the dangerous progression of atherosclerosis by reducing plasma cholesterol, decreasing plaque area and inhibiting lipid droplets accumulation. Similarly, apigenin treatment reduced plasma lipid levels and alleviated hepatic histopathological injury of TWR induced. It suggests the possibility of apigenin as a new therapeutic agent for the treatment of atherosclerosis. Notably, the effect of apigenin on inhibiting atherosclerosis has not exhibited dose-dependent effect. 12.5 mg/kg/d and 6.25 mg/kg/d apigenin can achieve better therapeutic effects. Therefore, in subsequent experiments, we will try to use a lower concentration gradient of apigenin to treat atherosclerosis.

RCT is the process of removing excess cholesterol from peripheral tissues to the liver (Zheng et al. 2022). The process is comprised of the cholesterol efflux from macrophage, extraction by liver into cholesteryl ester, then transfer to the bile and excretion into feces (Ohashi et al. 2005). Specifically, the accumulated cholesterol can be transferred from macrophages by ABCA1, ABCG5 and CD36 to peripheral circulation (Yu et al. 2013). In our study, HFD and TWR suppressed expression of ABCA1, ABCG5 and CD36, suggesting the high content of serum lipid is partially caused by impaired RCT. Furthermore, the ‘bad’ cholesterol LDL from the blood is delivered back to the liver *via* SR-BI, and eliminated through the gastrointestinal tract to maintain lipid homeostasis *in vivo* (Ohashi et al. 2005; Truong et al. 2010). Our results shown that apigenin remarkably upregulated the expression of lipoprotein receptors for delivering lipid, including SR-BI, ABCA1 and CD36. Based on this data, it is speculated that the lipid-lowering effect of apigenin is highly associated with increasing cholesterol efflux and promoting RCT, which is contributing to reduce atherosclerosis risks.

Autophagy involving in degradation of lipid droplets is termed as ‘lipophagy’. As a selective autophagy, lipophagy specifically targets and clears excess lipid in the liver (Omrane et al. 2023). The process of lipophagy involves the formation of autophagosomes, which is mediated by a series of autophagy-promoting genes (Luo et al. 2018; Xu et al. 2022). Beclin-1 functions as a platform by binding to UVRAG and PI3K complex to assemble the PI3KC3 complex during initiation of autophagosome formation (Wang 2015). The E3 ubiquitin ligase ATG5 forms a complex with ATG3 and ATG14 to promote autophagosome elongation (Lorincz et al. 2014; Danish et al. 2024). Herein, our data revealed that apigenin increased UVRAG, Beclin-1, ATG5 and ATG3 protein levels, up-regulated the mRNA expression of LC3B and ATG14 in hepatocyte of ApoE^−/−^ mice, indicating that apigenin treatment induced the increasing of autophagy flux. In addition, the lipase reduces lipid droplet size to the point in which they can fit into autophagosomes in the liver. Subsequently, the membrane proteins are recognized and sequestered by LC3II protein for the fusion of autophagosomes and lipid droplets (Omrane et al. 2023). Meanwhile the ATGs interact with ULK1 to drive these autophagosomes fusing with lysosomes to form autolysosomes, where the engulfed lipid droplets are degraded by lysosomal hydrolytic enzymes (Liu et al. 2020). In the present study, apigenin increased LC3II and ULK1 protein levels and activates autophagy (lipophagy), Moreover, the electron microscopy data provided support for the ability of apigenin promoting the formation of autophagosomes. We found that enhanced autophagy was associated with improved lipid accumulation from hepatocytes, which was consistent with previous reports (Leng et al. 2016). It was worth noting that we used immunofluorescence confocal microscopy to observe co-localization of UVRAG/LC3B and ADRP in hepatocytes. ADRP is often used as a marker of lipid droplet. The overlap between UVRAG/LC3B and ADRP indicates that autophagy induced by apigenin brings ADRP into the lumen of autophagic vacuoles for degradation, thereby reducing lipid droplets in hepatocytes. These collective results show that apigenin activates autophagy and lipophagy to regulate lipid metabolism, which has potential value in attenuating the formation of atherosclerotic plaques.

As a potential mechanism study for apigenin effects on autophagy, we further use PI3K inhibitor LY294002 to study the role of autophagy deficiency in TWR-induced mice. LY294002 can inhibit autophagy by preventing the association of Beclin-1 with the class I PI3K complex (Tan et al. 2019). We found that suppression of autophagy *in vivo* using LY294002, in part, abrogated the beneficial effects of apigenin during the initiation of atherosclerosis. Apigenin-induced changes in expression of blood lipid profiles (TC, TG, LDL-C), could be reversed by LY294002 treatment. Therefore, we surmise that apigenin mediates the activation of autophagy to encumber lipid accumulation. Sabrina Robichaud et al. have used yeast lipophagy assays to test several candidate lipophagy factors, including UVRAG and LC3B, finding a significant role for these proteins in lipid droplet catabolism (Robichaud et al. 2021). Our results found that apigenin could promote the expression of UVRAG and LC3B, while the expressions of lipophagy-related mRNA were decreased after LY294002 treatment. Additionally, the expressions of ABCG1, CD36 and SR-BI were decreased by LY294002, which indicated the association between lipophagy and lipid metabolism. Currently, the studies of lipophagy in atherosclerosis mainly focuses on macrophages. However, the studies of lipophagy in hepatocytes are relatively few. In this study,the lipid-lowering effects of apigenin are mediated through selective lipophagy in hepatocytes, while these effects are reversed by LY294002.

## Conclusions

In this study, we demonstrated that apigenin could favorably modulate serum and hepatic lipid contents, and reduce the formation of thoracic aorta and heart plaque in mice, which attenuated the initiation of atherosclerosis. Further mechanism study found that apigenin promoted RCT and increased the formation of autophagosomes. Moreover, the protective effect of apigenin was associated with enhancing UVRAG/LC3B-dependent selective lipophagy. In conclusion, our findings provide insights into the possible mechanism underlying apigenin-induced lipophagy; meanwhile, it provides a potential therapeutic option for atherosclerosis.

## Data Availability

The datasets generated and analyzed during the current study are available from the corresponding author upon reasonable request.
